# Leadership and management training as a catalyst to health system strengthening in low-income settings: Evidence from implementation of the Zambia Management and Leadership course for district health managers in Zambia

**DOI:** 10.1371/journal.pone.0174536

**Published:** 2017-07-25

**Authors:** Wilbroad Mutale, Anne-Thora Vardoy-Mutale, Arthur Kachemba, Roman Mukendi, Kupela Clarke, Dennis Mulenga

**Affiliations:** 1 University of Zambia School of Medicine, Department of Public Health, Lusaka, Zambia; 2 University of Zambia, School of Humanities, Department of Development Studies, Lusaka, Zambia; 3 Broachreach Institute for Training and Education (BRITE), Lusaka, Zambia; 4 Ministry of Health, Lusaka, Zambia; University of South Australia, AUSTRALIA

## Abstract

**Background:**

Research has shown that the modes of leadership and management may influence health outcomes. However, majority of health leaders and managers in many low-income countries are promoted on account of clinical expertise. It has been recognised that these new managers are often ill-prepared for managing complex health systems. In response to this challenge, the Zambian Ministry of Health (MoH) has developed the Governance and Management Capacity Building (GMCB) Strategic Plan (2012–2016), whose overarching goal is to improve health sector governance and create an environment that is result-oriented, accountable and transparent. This led to the introduction of a new in-service leadership and management course, which has come to be known as the Zambia Management and Leadership Academy (ZMLA). This paper presents the results of an impact evaluation of the ZMLA programme conducted in 2014.

**Methods:**

This was a cross-sectional mixed method study. The study targeted health workers, stakeholders and course implementers. ZMLA trainees were targeted to gain perspectives on the extent to which the programme affected levels of self-confidence resulting from knowledge gained. Perspectives were sought from both ZMLA and non ZMLA trainees to measure changes in the work environment. Stakeholder perspectives were collected from trainers and key informants involved in providing ZMLA training.

**Results:**

On average, knowledge levels increased by 38% after each workshop. A comparison of the average self-rated scores from 444 management and leadership survey responses before ZMLA and after ZMLA training showed a significant increase in the proportion of participants that felt adequately trained to undertake management and leadership, from 63% (before) to 99% (after) in phase 1 and 43% (before) to 98% (after) in the phase II cohort. The calculated before and after percentage change for work environment themes ranged from 5.8% to 13.4%. Majority of respondents perceived improvements in the workplace environment, especially in handling human resource management matters. The smallest improvement was noted in ethics and accountability. Qualitative interviews showed improvements in the meeting culture and a greater appreciation for the importance of meetings. Shared vision, teamwork and coordination seemed to have improved more in work places where the overall manager had received ZMLA training.

**Conclusion:**

Leadership and management training will be a key ingredient in health system strengthening in low-income settings. The ZMLA model was found to be acceptable and effective in improving knowledge and skills for health system managers with minimal disruption to health services.

## Introduction

Health systems governance is currently a critical concern in many countries because of increasing demand to demonstrate accountability in the health sector[1–3]. Research has shown that the modes of leadership and management may influence health outcomes such as life expectancy at birth, child mortality, maternal mortality, and self-reported health status.[4]. A study conducted in Kenya demonstrated increased coverage of maternal and child health services after piloting a six month leadership training in 67 management teams in Kenya [5].

It has been acknowledged that majority of health leaders and managers in developing countries are trained health professionals (doctors, nurses, clinical/medical officers and pharmacists) who rarely have any training or experience prior to being offered managerial positions[6, 7]. New managers are often promoted on account of clinical expertise alone. They may be ill-prepared for their new responsibilities and may be expected to gain managerial capacities by learning on the job or through training [6]. In the case of Zambia, district health teams are led by newly qualified medical doctors whose curriculum emphasizes clinical skills, with minimal attention to management and leadership. Recently there have been calls to strengthen leadership in the health sector through both in-service and pre-service training [1].

**Fig 1:**
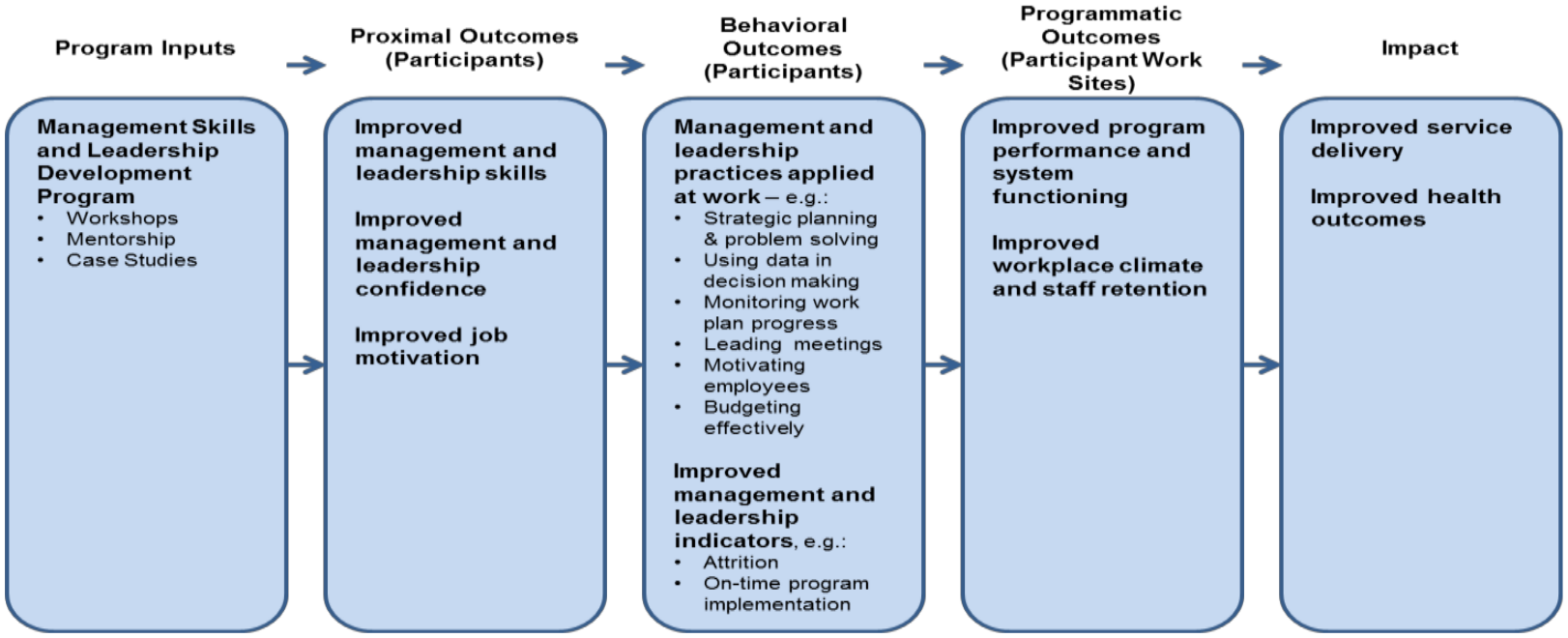
Cascade effect of the leadership and management training.

While didactic educational processes can be useful for providing ideas, material and motivation, they are not sufficient. The extent to which leadership and management are integrated into the personal identity depends on the extent to which the skills and knowledge are integrated into daily practice. This is often reinforced through coaching and mentoring[1]. Well integrated management and leadership practices can translate into positive change in organizational culture and routine processes. This can lead to improved performance which for health can be both system and health outcomes [4, 8]. This is summarised in the following logic framework:

The logic model (Fig 1) combines leadership and management concepts from Donald and James Kirkpatrick's learning and training evaluation theory,[9] McLeroy et al (1988) socio-ecological model of behavioural[10] and work done by the Management Sciences for Health (MSH). The model suggests that through exposure to program activities (workshops, mentoring, case studies), program trainees should:

- Demonstrate increased knowledge
- Be more confident in carrying out management and leadership functions
- Experience improved job motivation
- Improve management skills and behaviours that increase daily work performance.

This conceptual framework informed the design and evaluation of the ZMLA Programme. It was expected that participants exposed to the ZMLA programme would show improvements in the areas of strategic planning, problem analysis, use of data in decision making, running meetings, supervising and motivating employees. As a result of these changes in participants’ work practices, programme performance/service delivery and the work climate at participants’ work sites should also improve. By improving programme performance and service delivery, it was anticipated that the ZMLA programme would ultimately benefit the general population through access to better health services [11]. Complementing the logic model was the McLeroy et al (1988) socio-ecological model of behaviour change, which posits that behaviour is determined by the following factors:

- Personal factors: Characteristics of individuals, which include knowledge, attitude, behaviour, self-perceptions
- Institutional factors: These include social institutions, rules, and other organizational characteristics
- Community factors: Relationships among organisations, institutions, individuals, cultural systems, beliefs.
- Public laws and policies: For example national policies

It was theorised that improved skills and adoption of good practices would lead to improved workplace environment, improved performance and service delivery. This model was adopted as it captured the activities and aims of the leadership training course presented in this paper.

In view of the current leadership crisis in the health sector, the Zambian Ministry of Health (MOH) had developed the Governance and Management Capacity Building (GMCB) Strategic Plan (2012–2016), whose overarching goal was to improve health sector governance and create an environment that is result oriented, accountable and transparent. This led to the adoption of a new in-service leadership and management course, which has come to be known as the Zambia Management and Leadership Academy (ZMLA). The course has been adapted for the Zambian health system managers. The development of the course was supported by the Ministry of Health (MOH), Ministry of Community Development, Mother and Child Health (MCDMCH), BroadReach Institute for Training and Education (BRITE) and the Zambia Integrated Health Systems Strengthening Project (ZISSP). The course was accredited by the National Institute of Public Administration (NIPA). It was generally conducted over a period of 6–12 months. It had both theoretical and practical sessions which were supported by mentorship both during and after training. The training was delivered in four (4) workshops and the content arranged in six (6) modules. (Table 1)

**Table 1:** Summary of ZMLA course content.

Workshop #	Content delivered over 2–2.5 day for each Workshop	
**1**	**Module 1: Problem Definition** Problem definition Strategic and operating planning Strategic planning frameworks Relevance of strategic planning to the organization Problem Analysis Tools Prioritization Critical Thinking and Pressure Testing Critical Path	**Module 2: Strategic Planning** Basics of supply chain management Value chains and implementation frameworks Model of care Developing implementation plans and Work plans Internal and external stakeholder identification and analysis Bottlenecks and the Marginal Budgeting for Bottlenecks Planning process in Zambia Performance assessments Zambia planning resources (tools and guidelines)
**2**	**Module 3: Project Management Fundamentals** Organizational structures and charts Definition of a project, project management and project manager role Defining project success Leadership versus. Management Running and participating in meetings	Delegation Decision rights Teamwork Providing useful feedback Change management
**3**	**Module 4: HR Management** Employee lifecycle Recruitment and retention strategies Incentive systems and motivation tools HR Development Performance management–supervision, discipline and appraisal)	**Module 5: Finance Management** Financial management overview Budgeting, forecasting, and reporting Cost management Financial statements and reporting
**4**	**Module 6: Strategic Information management** M&E as management tool M&E approach M&E metrics Building information culture Using and maintaining information systems	

The ZMLA course has been packaged in line with a recent study that recommended experimentation with action learning approaches, including a mix of formal training, on-the-job training, mentoring and support. So far, the ZMLA training has been offered to over 700 health workers in 27 districts between 2012–2014. The overall objective of the programme was to improve participants’ knowledge of management and leadership skills and help them employ these in their day-to-day duties. The first phase of the program was completed in September 2013; the second phase concluded a year later in September 2014. The third phase of 116 trainees completed the program in December 2014 (Table 2).

**Table 2:** Recruitment and Completion Dates for ZMLA implementation phases.

	Enrollment and Completion Dates	Number of Trainees Enrolled
**Phase 1**	Oct 2011 –Jun 2013	474
**Phase 2**	Dec 2013 –June 2014	177
**Phase 3**	Jul 2014 –Dec 2014	116

This paper presents results from the programme evaluation, which aimed to measure the extent to which the ZMLA training had contributed to improved management and leadership skills and working environment in line with the ZMLA conceptual framework, in addition to sharing lessons learnt through implementation of the course over a 4-year period.

## Methodology

### Study design

This was a cross-sectional mixed method study. Both qualitative and quantitative methods were used to collect information.

### Target population

The study targeted health workers, stakeholders and course implementers. ZMLA trainees were targeted to gain perspectives on the extent to which the program affected levels of self-confidence resulting from knowledge gained. Perspectives were sought from both ZMLA and non-ZMLA trainees to measure changes in the work environment. Stakeholder perspectives were collected from trainers, and key informants involved in providing ZMLA training.

### Study sites

The study was conducted in all 10 provinces of Zambia and selected target districts.

### Eligibility criteria

All participants who took part in the training were eligible for the pre-post training interview. Those that had not completed both pre-post training questions were not included in the final analysis.

The workplace survey questionnaire was only administered to worksites of phase II. Phase I participants did not fill in the workplace survey at baseline.

Health workers found at the targeted workplace were eligible regardless of exposure to the ZMLA training. Key informant interview, included those working in their capacity as provincial, district and health facility managers regardless of the time they had been in the position. Those acting where interviews if the substantive respondent was absent during data collection.

### Sampling

All participants for phases I and II were given a pre- and post-training questionnaire to assess changes in knowledge and skills. Eight (8) out of twenty-seven (27) districts were purposefully sampled to take part in the workplace survey. Qualitative data was collected from 70 key informant interviews (Table 2). The selection of respondents was purposeful and based on their positions within their organizations and their experience with the ZMLA program. Efforts were made to ensure that key informants from all stakeholders were represented. Each of the 10 provincial health offices was visited for key informant interviews.

### Data collection

Routine data collection tools were used to monitor ZMLA progress and program quality. Knowledge quizzes were designed to test participants’ knowledge on several concepts before and after being trained. Participant feedback forms were collected after each training session. Participants were asked to develop group and individual case studies. The quality of all case study plans developed by participants was submitted to NIPA to assess participant’s ability to apply concepts learned.

## Management and leadership survey

The management and leadership survey was used to measure changes in confidence and job motivation among ZMLA trainee respondents. The tool was administered twice to 164 phase I and to 280 phase II ZMLA trainees at the beginning and at the conclusion of each training. The self-perception confidence questions in the survey were premised on Albert Bandura’s concept of self-efficacy, which posits that a person who feels greater confidence in his or her ability to perform a specific behaviour is more likely to successfully perform the behaviour. The management and leadership tool used a 10-point Likert scale to measure responses to statements relating to management capacities, and a 5-point Likert scale to measure responses to statements relating to job-satisfaction and commitment to the organization. The questions were adapted from the Clinical Leadership Competency Framework (CLCF) and Medical Leadership Competency Framework (MLCF) of the United Kingdom. (www.leadershipacademy.nhs.uk). The tool was piloted and before being used in the current study. Before conducting the pilot, we first had consultative meetings with the Ministry of Health Technical Services team under which the Leadership development for MoH is hosted to review the tool. We also had consultative meetings with other stakeholder to contextualise and adapt questions to reflect the Zambian health care contexts. In addition, we reviewed the questions to ensure alignment with ZMLA course content which was the basis for the final evaluation. The draft tool was then administered to a sample of health workers in Lusaka who were not part of the ZMLA training. The responses were reviewed and adaptations were made based on pilot experience to come up with the final tool.

## Workplace climate tool

The workplace survey questionnaire was only administered to worksites of phase II trainees that were trained from December 2013 to June 2014. Phase I participants did not fill in the workplace survey at baseline, making it inappropriate to use the data to measure perceived changes without relocation biases. Phase I data was thus not used in this evaluation.

The workplace climate survey was designed to measure existing conditions and changes in the workplace environment as scored before and after the course. The survey targeted everyone at a workplace, regardless of exposure to the ZMLA course.

The survey was self-administered with a ten-point Likert scale measuring personal perceptions of the work environment. The survey was administered before the start of the ZMLA trainings and shortly after the conclusion of the training (one to three months) in nine randomly selected districts health offices. Unlike the management and leadership survey, there was no pairing of respondents at baseline and end line evaluation and the data set had no individual identifiers to track individual responses at baseline and end line. The tool was adapted from the work climate review paper by Gershon,R et al 2004 [12].

## Qualitative data collection

In-depth interviews with key informants were conducted in target provinces and districts. The selection of participants was purposeful. However, efforts were made to ensure representation from target districts and provinces. The main respondents were provincial medical officers, district medical officers, hospital administrators, ZMLA course implementers, mentors and selected stakeholders. Additionally, for each provincial office and selected district offices, 1–2 people trained in ZMLA were selected to take part in the study. We used an interview guide to collect the data. This was a flexible tool allowing for exploring issues as they arose during the interviews.

## Data management

Quantitative data was entered into access database and exported to SPSS for analysis. Data was cleaned and all outliers were validated by checking against hard copy entries. Qualitative data was recorded using digital recorders. It was downloaded and backed up at the end of each day and later transcribed by trained research assistants.

## Analysis of quantitative data

Quantitative data was exported to SPSS version 19 for analysis. Simple frequencies were used to explore the data. The basic unit of analysis was individual participants. Chi-square was used to compare between categorical variables and t-test to compare continuous variables. Statistical significance was set at 95% confidence level.

## Qualitative data analysis

Trained research assistants experienced with qualitative methods transcribed data. After data cleaning, transcripts were exported into QSRNVivo 10 for analysis. Two of the authors (WM and AT) reviewed transcripts of interviews, validating pre-determined themes and identifying additional themes and subthemes that emerged. These themes where validated with other co-authors who confirmed the adequacy of the themes and subthemes. Two of the authors coded the data. In order to ensure consistency in data coding, we used Cohen Kappa, which is in-built into the Nvivo Software to check for inter-coder reliability[13]. This was found to be 0.72, corresponding to substantial agreement in coding by the two coders[13]. The pre-determined themes were based on the course contents and objectives (Table 1). Thematic analysis was then employed focusing on identifying patterned meaning across the data. We compared themes by districts, level of care, category of health workers and attendance of the ZMLA in order to understand the different perspectives of the ZMLA course.

## Ethical consideration

The study obtained permission from the University of Zambia Biomedical Ethics Committee and the Ministry of Health. All participants who responded to the surveys were asked to provide written consent. All data were stored securely in a password protected Access database. All information was anonymised and no personal identifiers were used during analysis of data and publication.

## Results

### Demographic characteristics of participants

For key informant interviews, a total of 70 respondents were interviewed. Thirty (30) participants were from Provincial health office, twenty-four (24) participants were from the district teams, seven (7) and six (6) respectively were from the national level and health facility. Majority of the participants were medical doctors at both district and provincial level. (Table 3)

**Table 3:** Public health system level and job categories of key informants interviewed.

Public Health Systems Level	Job Category	Number
**District**	Case Study Participant	5
	Clinical Care Specialist	1
	District Medical Officer	9
	Finance Officer	1
	Health Management Information Officer	1
	Hospital Administrator	1
	Human Resource Officer	2
	Medical Superintendent	1
	NGO Coordinator	1
	Planner	2
	Public Health Officer	2
	**Sub Total**	**24**
**Health Facility**	Health Facility Manager	4
	Nursing Officer	2
	**Sub Total**	**6**
**National**	MOH Director	1
	Trainer	2
	ZMLA Implementer	5
	**Sub Total**	**7**
**Provincial**	Clinical Care Specialist	1
	Finance	3
	Human Resource Officer	2
	Management Specialist	8
	Nursing Officer	2
	Planner	3
	Provincial Medical Officer	8
	Principal Dental Therapist	1
	Public Health	2
	**Sub Total**	**30**
	**Grand Total**	**70**

For the leadership and management questionnaire, 280 participants were interviewed in the phase 1 while 164 were interviewed from phase 2 giving a total of 444 participants. The majority were males (70%) and urban residents (59%) (Table 4).

**Table 4:** Demographic characteristics of leadership and management survey respondents.

Demographic Characteristic		Phase I	Phase II	Grand Total
**Public Health Systems Level**	Community	1	1	2
	District	101	104	205
	Facility	51	45	96
	National	18	3	21
	Provincial	109	11	120
**Residence**	Rural	82	101	183
	Urban	198	63	261
**Gender**	Female	85	48	133
	Male	195	116	311
**Job Category**	Administrative	66	46	112
	Managerial	26	24	50
	Technical Medical	94	56	150
	Technical Non-Medical	75	37	112
	Traditional Leader	1	1	2
	Not Categorized	18		18
	**Total**:	280	164	**444**

### Knowledge change pre and post workshop

**Fig 2:**
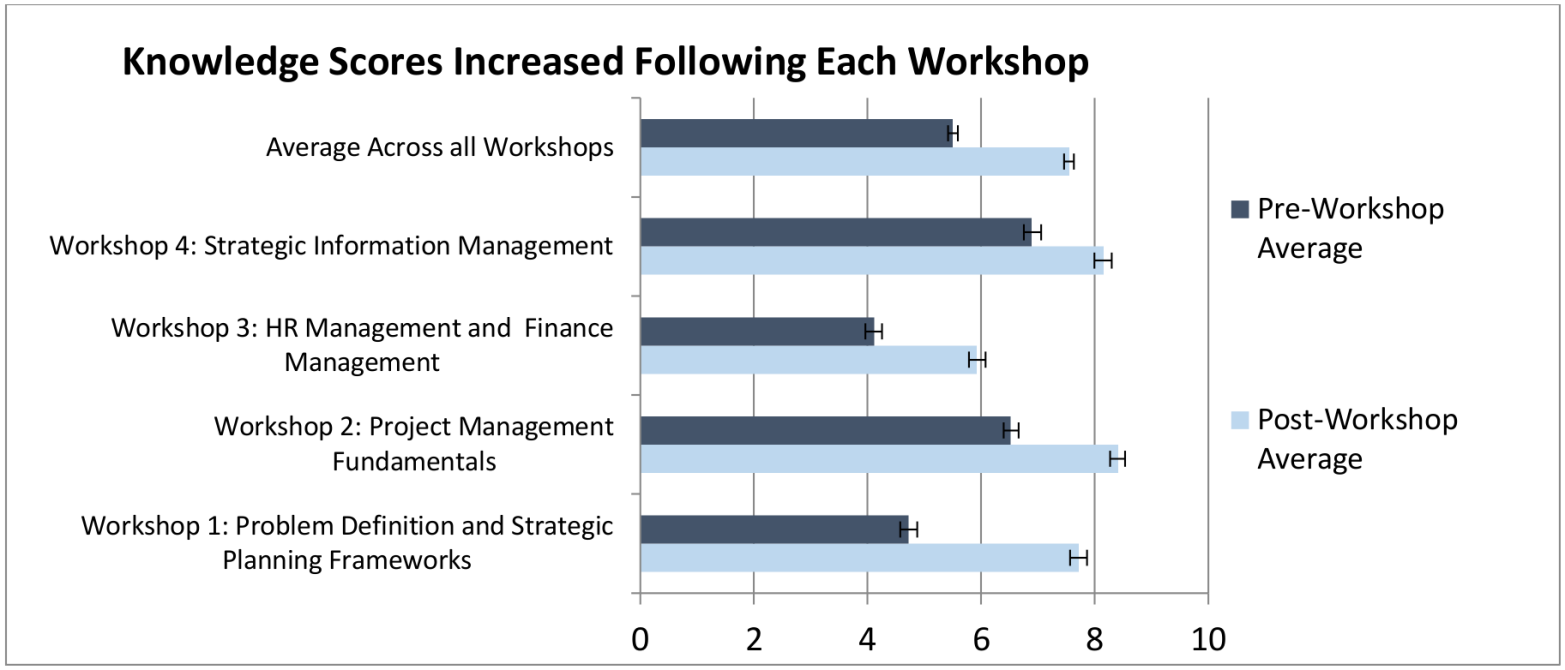
Knowledge scores pre & post workshops.

On average, knowledge levels increased by 2.1 points (38%) after each workshop. The most significant change in terms of gain in knowledge was noted for workshops 1 and 2, by 3.0 (63%) and 1.9 (29%) points respectively. Workshops 4 showed the least change in skills score 1.3 (19%) points (Fig 2).

### Case study performance by trainees

To supplement individual knowledge assessments through pre- and post-test quizzes, the programme required participants to demonstrate their understanding of various ZMLA concepts by applying them to practical case studies. The results indicated that participants performed best in the area of creating relevant and sound monitoring and evaluation frameworks (93%), but had difficulties in developing a model of care illustration (58%), delegation plan (56%), and job descriptions (50%) (Table 5).

**Table 5:** Case study scoring.

Scoring Factors	Avg. Score	Total Points Possible	%
M&E Framework	27.9	30	93%
Direct and cross-cutting functions listed	4.1	5	83%
SMART objectives developed	3.9	5	78%
Work plan/ Gantt Chart	3.8	5	75%
Solutions identified and prioritized	3.7	5	74%
Strategic planning and problem tools have been applied to ID root causes	3.7	5	73%
Decisions rights matrix	3.6	5	73%
Organizational structure for the project	3.6	5	72%
Budget	3.6	5	72%
Model of care illustration	2.9	5	58%
Delegation Plan	5.6	10	56%
Job description clearly defined for all key players in the organizational structure	5.0	10	50%
TOTAL	71.4	95	75%
N			72

### Leadership and management skills

**Fig 3:**
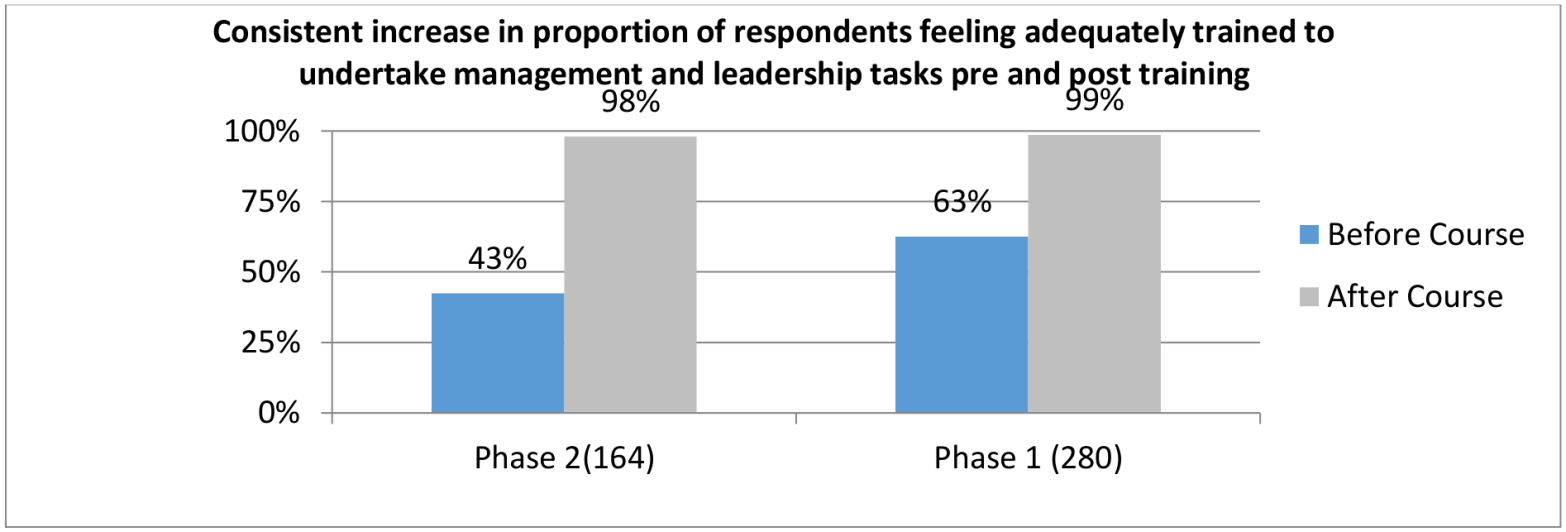
Self-Assessment of ZMLA trainee management and leadership readiness.

To understand how the training further improved individual leadership and management skills, both qualitative and quantitative methods were used. A comparison of the average self-rated scores from 444 management and leadership survey responses before ZMLA and after ZMLA training showed a significant increase in the proportion of participants that felt adequately trained to undertake management and leadership: Phase I from 63% before to 99% after, Phase II from 43% before to 98% after (Fig 3).

**Fig 4:**
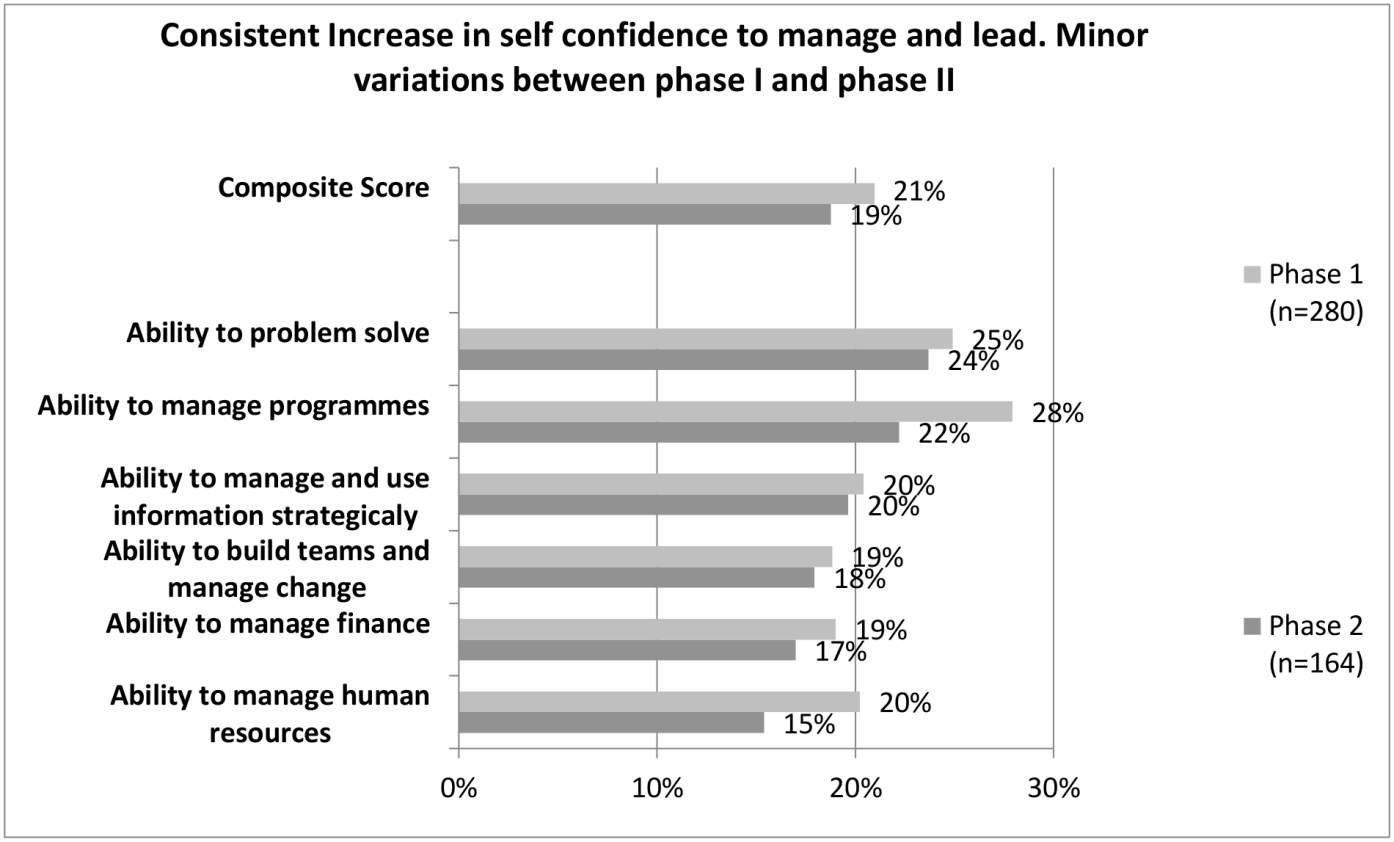
Percentage change in management and leadership self confidence.

In addition to participants feeling adequately trained after undergoing the training, an assessment of self-perceived improvement in undertaking a range of management and leadership tasks, significantly improved. Fig 4 highlights self-perceived percentage changes in mean scores for six broad management and leadership tasks. The greatest changes were noted under the themes ‘problem solving, planning and managing programmes. The differences across all themes were statistically significant for all variables in the leadership and management tool.

### Individual items change before and after ZMLA training

Individual item analysis for the 22 items on the leadership and management scores indicated that generally the baseline scores were high with average scores all above 7/10 for the first 22 items. The follow-up scores ranged between 8–9 points out of 10 for the 22 items. Result showed that there were improvements in leadership and management scores after the ZMLA training across all the 22 items. The differences were all statistically different for all the 22 items in the leadership and management tool. The highest mean score change was in the item relating to analyzing the model of care for health services to identify major bottlenecks. This was followed by the related item, which referred to the capacity to address gaps identified after problem analysis (Table 6). These findings validated results from the qualitative assessment, which showed that most participants appreciated the problem analysis so they could get to the root causes of problems (atomisation).

**Table 6:** Leadership and management score before and after ZMLA training.

	N	Before	After
1. Provide constructive feedback on a regular basis, in a way that helps those I supervise improve their performance.	156	7.85 (SD: 1.71):	8.92 (SD: 1.32)
2. Use non-monetary strategies (such as praise, public recognition, and reminding staff of the importance/value of their work) to motivate those I supervise.	155	7.81(SD: 1.81)	8.98(SD: 1.31)
3. Identify staff development needs and work with them to plan appropriate trainings, mentoring opportunities, or other ways of addressing the needs.	144	6.85 (SD: 2.03)	8.41 (SD: 1.47)
4. Chair productive and efficient meetings that begin and end as scheduled.	148	7.89 (SD: 1.73)	9.01 (SD: 1.23)
5. Identify the most important “root” or underlying causes of specific challenges within my unit/department that affect the healthcare system (such as high maternal mortality, or ART non-adherence).	135	7.41 (SD: 1.92)	8.94 (SD: 1.13)
6. Prioritize among possible solutions/interventions to address healthcare challenges, to identify and implement those that will have the highest impact with fewer resources.	147	7.18 (SD: 1.85)	8.65 (SD: 1.42)
7. Engage and maintain effective communication with internal and external stakeholders (including other departments or levels within the MOH, community members/organizations, NGOs, and other ministries) when planning and implementing strategies to address	146	7.10 (SD: 1.78)	8.51(SD: 1.30)
8. Develop detailed workplans that specify timelines, milestones, and roles/responsibilities of specific people, to address specific challenges or achieve certain goals (such as increasing use of family planning services, improving access to EMoC,	150	7.02 (SD: 1.98)	8.73 (SD: 1.25)
9. Regularly check in on progress, and hold members of my organization/unit accountable for following through on objectives and activities from our workplan or Action Plan.	154	7.15(SD: 1.86)	8.82(SD: 1.32)
10. Ensure that projects I oversee are carried out within the allotted budget, time and resources.	152	7.88(SD: 1.68)	8.93(SD: 1.18)
11. Promote teamwork and collaboration among staff and different units in my organization.	156	8.19 (SD: 1.55)	9.16 (SD: 1.08)
12. Communicate well with staff, to ensure that they understand the overall picture (strategic vision) of our unit and1 are informed about any changes that may be introduced to our organization.	155	7.942 (SD: 1.75)	9.14 (SD: 1.05)
13. Follow up to facilitate prompt resolution of staff HR issues, such as promotion and training.	107	7.22 (SD: 1.97)	8.54 (SD: 1.30)
14. Ensure that staff reporting to me have up-to-date job descriptions, with which they are familiar, and understand reporting lines within our unit/department.	131	7.40 (SD: 1.96)	9.01(SD: 1.960)
15. Take appropriate corrective action to address staff performance problems as soon as I am aware of them.	136	7.574(SD: 1.64)	8.94 (SD: 1.21)
16. Identify and apply cost-effective approaches to maximize use of the organization’s resources.	155	7.81(SD: 1.69)	8.95 (SD: 1.24)
17. Create realistic programme or project budgets based on historical data, current cost information, and other relevant information sources.	147	7.51 (SD: 1.94)	8.70 (SD: 1.35)
18. Effectively interpret and use the data available to me (such as from HMIS, performance assessment reports, and other sources) to guide planning, decision-making, and quality improvement.	155	7.61(SD: 1.93)	8.95 (SD1.31)
19. Create reports, charts and graphs which succinctly and effectively communicate relevant data to stakeholders.	147	7.25(SD: 2.15)	8.82 (SD: 1.28)
20. Analyze the delivery model for health services to identify the major gaps and bottlenecks affecting the quality of care and health outcomes.	144	6.55 (SD: 2.12)	8.42 (SD: 1.39)
21. Identify and implement necessary changes in delivery models for health services to address or correct gaps and bottlenecks affecting quality of care and health outcomes.	137	6.55(SD: 8.28)	8.28 (SD: 1.48)
22. Use lead times, inventories, and other supply logistics information to ensure that stock levels of critical supplies remain adequate at all times.	129	6.77 (SD: 1.91)	8.43 (SD: 1.46)
**Total mean leadership and management scores**		**7.39**	**8.37**
**Percentage change before and after ZMLA training**			**11%**

Note: P<0.05, before and After for all the 22 items and overall score

Qualitative findings complement the above quantitative results. In interviews, 55% (34/62) of the eligible respondents reported that they felt more prepared and motivated to perform their duties after their training:

“Work is a bit more enjoyable now because I know exactly what I need to do. So I have a reason to get up and go to the office because there is something that I have written down somewhere that this is what I need to do. Before it was very difficult for us to come up with work plans. It was like Greek, but now I know that I need to do this so that I can get that done, you plan your day well in advance because you know there’s something that you want. So I guess I’m more alive when I go to work now than going to work and thinking what are we going to do now.”

ZMLA trainee, Mbala

As an example, trainees felt confident in preparing their annual action plans. Trainees no longer copied and pasted plans from previous years but developed original action plans with competence and efficiency.

“…If you look at what we do, it’s mainly to do with planning. Like right now we are doing planning, we are developing our work plans, we look at the medium term expenditure framework going into the strategic plans…all those things that we were learning in ZMLA….,now we are not scared to go for planning…we now know how it should be done and my team is ready.”

ZMLA trainee, Nchelenge

### Job motivation outcomes

To measure improvements in job motivation before and after undergoing ZMLA training, a series of questions were asked in the management and leadership survey. The focus of the assessment was: (i) commitment to career in the organization, (ii) optimism about future success in the organization, (iii) enjoying tackling new challenges arising in work, (iv) and feelings of positivity while working.

**Fig 5:**
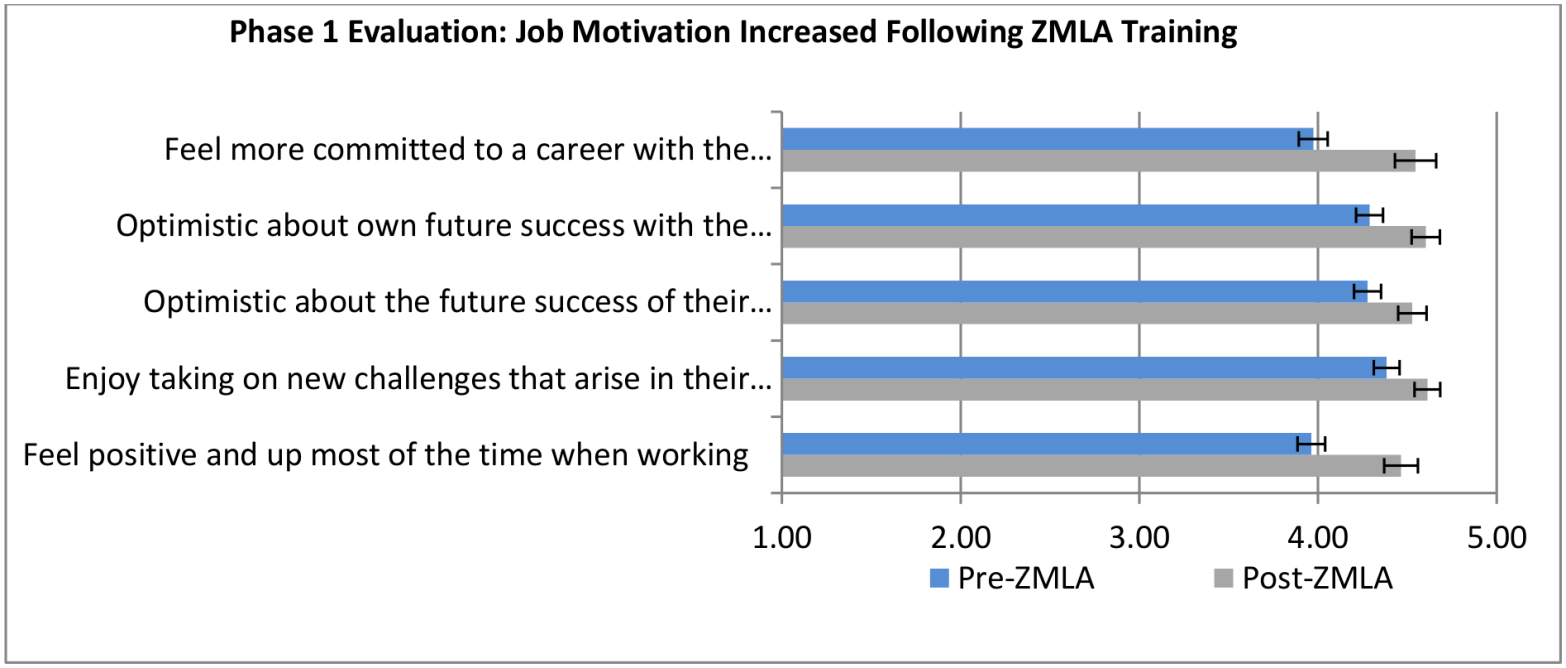
Phase 1 & 2 evaluation of job motivation.

Fig 5 shows mean differences and associated confidence intervals for a 5-point Likert scale of before and after training. Commitment to a career in their organization and feelings of positivity when at work were the most notable changes in trainee job motivation. These findings were consistent across phase I and phase II of training.

Key informant interviews revealed that the ZMLA training contributed to their decision to stay in the public health service. The reasons given by majority of the trainees (52%) were that they now felt empowered and confident to be managers in the health sector and that they felt more motivated with skills and knowledge gained from the ZMLA.

“Job motivation has improved, of course may be not in terms of remuneration but in terms of motivation, because when you know what you are supposed to do it becomes a motivation on its own.”

ZMLA trainee, Mansa

## Organizational benefits resulting from ZMLA trainings

### Changes in the workplace climate

The characteristics of work climate respondents are presented in Table 7.

**Table 7:** Work climate survey respondent characteristics.

	Demographic Characteristic	Pre-ZMLA	Post-ZMLA
District Medical Office	Chilubi	7	6
	Chongwe	7	10
	Lundazi	6	6
	Masaiti	16	9
	Nchelenge	7	6
	Shangombo	12	2
	Sinazongwe	9	8
	Zambezi	9	11
Job Category	Administrative	15	13
	Technical (clinical)	22	17
	Technical (non-clinical)	27	19
	Technical (public health)	9	9
ZMLA/None ZMLA	ZMLA Participant	47	36
	None ZMLA Participant	26	22
Gender	Female	13	14
	Male	60	44
	**Total**	**73**	**58**

In evaluating the organizational benefits that resulted from ZMLA trainings, quantitative data from the workplace climate survey and qualitative themes derived from 70 in-depth interviews were analysed.

The overall workplace climate improvement after ZMLA was by 5.3%. The highest change was reported by non-ZMLA trainees with 8.6% improvement. Workplace climate change reported by ZMLA trainees was 3.8% improvement. These results suggest that non-ZMLA trainees might be a good source of information on workplace climate as they were independently confirming the effect of ZMLA training (Table 8).

**Table 8:** Workplace climate assessment before and after ZMLA training.

	*Pre test*	*Post-test*	*Pre-test*	*Post-test*	*Pre-test*	*Post-test*
1. In this office employees understand the organizational structure and reporting lines of their unit/department, and how their job functions relates to overall departmental objectives and goals	7.56 (SD:2.39)	8.01 (SD: 2.17)	7.77 (SD: 2.12)	8.14 (SD: 2.21)	7.19 (SD:2.82)	7.81 (SD:2.12)
2. For most meetings called for in this office, agendas are circulated to all before the meeting.	7.17 (SD:2.89)	7.45 (SD: 2.51)	7.62 (SD:2.56)	7.58 (SD: 2.25)	6.38 (SD:3.29)	7.22 (SD:2.93)
3. For most meetings called for in this office, minutes are circulated to all soon after the meeting, indicating follow-up items.	5.39 (SD: 2.89)	6.07 (SD: 2.69)	5.93 (SD: 2.71)	6.64 (SD: 2.27)	4.42 (SD:2.99)	5.14 (SD:3.09)
4. The leadership here keeps staff well informed about what is going on with the organization.	7.52 (SD: 2.34)	8 (SD: 2.43)	8.1 (SD: 1.97)	8.14 (SD: 2.38)	6.46 (SD:2.76)	7.77 (SD:2.54)
5. In this office, cooperation and teamwork between staff in different units is encouraged.	8.26 (SD: 2.23)	8.14 (SD: 2.25)	8.79 (SD: 1.44)	8.25 (SD: 2.09)	7.31 (SD:3.00)	7.95 (SD:2.53)
6. In this office, we are encouraged to use data to guide decision-making, priority-setting, and planning.	7.58 (SD: 2.72)	8.31 (SD:2.31)	7.74 (SD: 2.44)	8.69 (SD: 1.92)	7.27 (SD:3.19)	7.68 (SD:2.77)
7. In this office, we are encouraged to analyze problems carefully to understand root causes before deciding on solutions.	7.48 (SD: 2.46)	8.1 (SD 2.24)	7.77 (SD: 1.86)	8.58 (SD: 1.86)	6.96 (SD:3.26)	7.32 (SD:2.60)
8. In this office formal individual performance appraisals are routinely conducted on an annual basis.	5.95 (SD: 2.79)	6.74 (SD: 2.84)	6.09 (SD: 2.59)	6.81 (SD: 2.99)	5.69 (SD:3.15)	6.63 (SD:2.62)
9. In this office supervisors provide constructive feedback to their assistants on a regular basis, to help improve job performance.	6.75 (SD: 2.56)	7.36 (SD: 2.31)	6.91 (SD: 2.19)	7.58 (SD: 2.18	6.46 (SD:3.12)	7.00 (SD:2.50)
10. My contributions at work are acknowledged and appreciated.	7.67 (SD: 2.44)	7.88 (SD: 2.30)	7.98 (SD: 1.95)	8.25 (SD: 1.93)	7.11 (SD:3.49)	7.27 (SD:2.75)
11. My supervisor works with me to identify my training needs and ensure I get the training or mentorship I need to do my job effectively.	6.97 (SD: 2.90)	7.14 (SD: 2.68)	7.4 (SD: 2.46)	7.19 (SD: 2.42)	6.19 (SD:2.91)	7.05 (SD:3.12)
12. At this office, when staff attend trainings, effort is made to ensure that they apply what they have learned back at the job site.	7.16 (SD: 2.48)	7.59 (SD: 2.22)	7.66 (SD: 2.08)	8.22 (SD: 1.88)	6.27 (SD:3.37)	6.55 (SD:2.39)
13. In this office supervisors delegate challenging assignments to assistants, which helps them to develop their skills and expertise.	7.29 (SD: 2.57)	7.69 (SD 2.01)	7.64 (SD: 1.95)	7.92 (SD: 1.93)	6.65 (SD:2.86)	7.23 (SD:2.11)
14. In this office when giving special assignments, supervisors clearly communicates expectations at the beginning and checks in on progress, without ‘micromanaging.’	6.98 (SD: 2.41)	7.17 (SD: 2.54)	7.34 (SD: 2.07)	7.47 (SD: 2.55)	6.34 (SD:3.18)	6.68 (SD:2.49)
15. In this office supervisors or unit leader regularly monitor progress and holds every staff accountable for following through on assigned tasks related to work plans.	7.3 (SD: 2.45)	7.12 (SD: 2.59)	7.62 (SD: 1.89)	7.36 (SD: 2.61)	6.73 (SD:3.59)	6.73 (SD:2.51)
16. In this office supervisors do everything in their power to help resolve HR issues (such as confirmation) in a timely manner.	**6.3** (SD: 3.12)	7.41* (SD: 2.12)	**6.72** (SD: 2.78)	7.64* (SD: 2.97)	**5.54** (SD:3.11)	7.05* (SD: 2.64)
17. In this office supervisors take appropriate corrective action when an employee is not performing well.	7.11 (SD: 2.5)	7.67 (SD: 2.29)	7.38 (SD: 2.22)	7.86 (SD:2.34)	6.62 (SD:2.91)	7.36* (SD:2.12)
18. In this office supervisors maintains a high standard of ethics and accountability.	7.97 (SD: 2.32)	7.72 (SD: 2.65)	8.23 (SD: 1.90)	7.75 (SD:2.15)	7.50 (SD:2.19)	7.68 (SD: 2.68)
*Mean*	*7*.*13* *(SD*: *2*.*71)*	*7*.*53* *(SD*: *2*.*27)*	*7*.*48* *(SD*: *2*.*38)*	*7*.*78* *(SD*: *2*.*77)*	*6*.*50* *(SD*:*2*.*32)*	*7*.*11* *SD*:*2*.*22)*
***Percentage change***		***5*.*28%***		***3*.*84%***		***8*.*61%***

Note:

* = P<0.005

**Fig 6:**
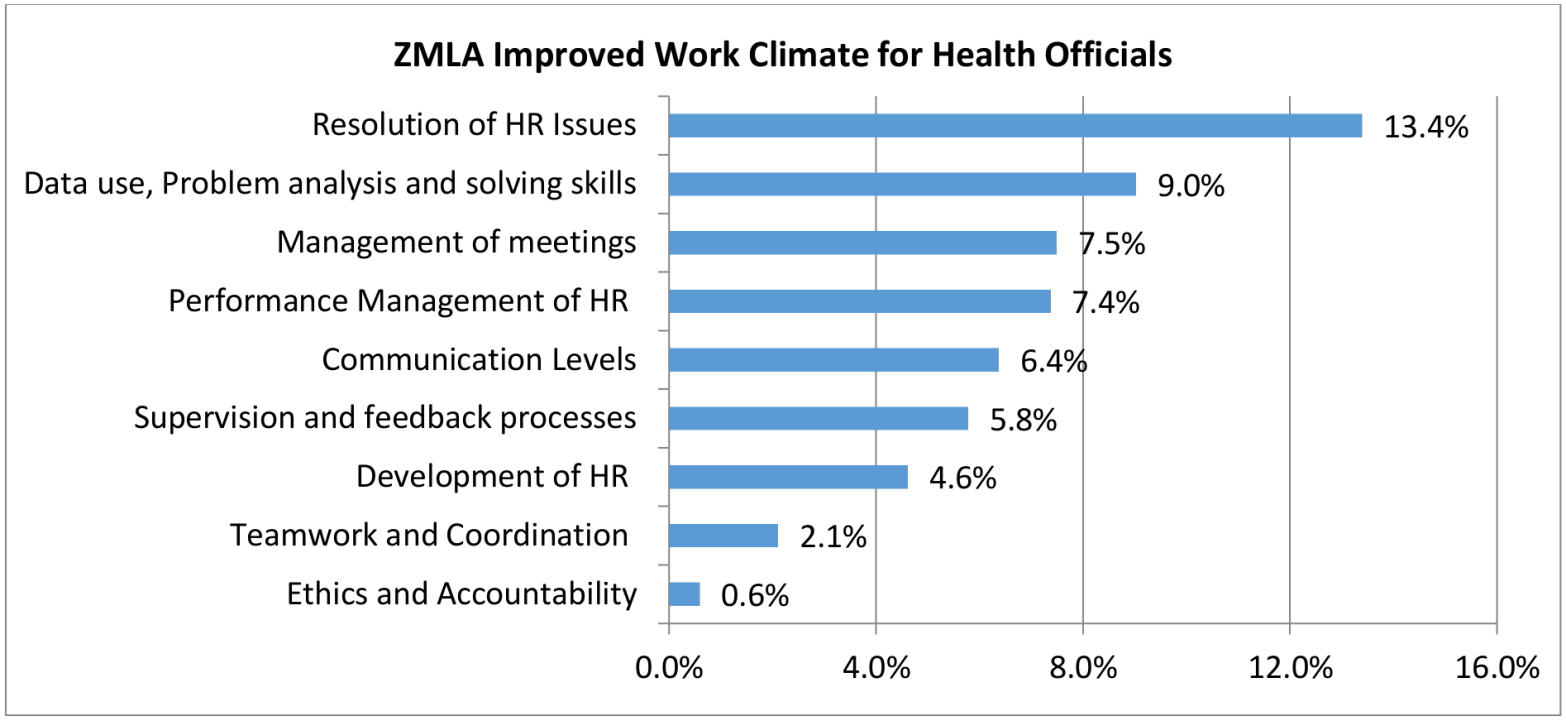
Evaluation of work climate survey.

Further analysis showed that respondents perceived improvements in the workplace environment especially in handling human resource management matters. The least improvement was noted in ethics and accountability. The calculated before and after percentage change for work environment themes ranged from 0.6%-13.4% (Fig 6)

## Overall conduct of ZMLA and lessons learnt

### Course duration

Participants from phase I of implementation were trained for a minimum of 12 months, which was seen as a very long time to complete the ZMLA course as explained by one respondent:

“I think our course was too long when you compare it to those who are doing it now. The course went on for over one year…it is very difficult for full-time workers to attend such a course…6 months is the ideal time for such a course.”

ZMLA trainee from phase I.

By the second phase of implementation, adjustments to the length of the course reduced the course to just six months. While this was seen as an improvement in the length of training, it was also associated with significant work overload for some of the trainees interviewed from the second phase of implementations. There was also a strong feeling that there was little time allocated for participants to go through practical examples and adequately share experiences. Many participants felt that adding another training day would make a big difference.

“In my view the course is overloaded…some of the concepts require more time. Some topics such as financial management need more time…but currently the time allocated is insufficient.”

Course implementer, Southern Province

“The time allocated for the course is too short. There are so many things to learn and some of the concepts are new to many participants…it would help even to add just an extra day.”

ZMLA trainee, Kalabo

### Participant commitment

Participants’ commitment to ZMLA was not as high in the first phase as in the second and third phases. ZMLA was not well known, and it was unclear whether the training would be certified. After ZMLA was certified by NIPA and became better known, participants’ enthusiasm for and commitment to the course increased.

“In the initial stages when they didn’t know what the training entailed, there was a bit of resistance and some people couldn’t complete the course. Now everyone wants to get ZMLA training because they have discovered that you get the knowledge and a certificate that you can use elsewhere.”

Course implementer, Northern Province

This observation was reinforced by the reduction of attrition from as much as 32% in the first phase to just 8% in the second phase of implementation. The high dropout of phase I participants was also addressed in phase II by merging workshop and mentorship sessions into the same week rather than spacing sessions four weeks apart.

From all interviews conducted, it was apparent that ZMLA was popular among participants. One reason given across all cadres was that ZMLA was filling an important gap in leadership and management knowledge and skills among health workers. The course was said to increase self-confidence in leaders and provided them with practical tools to address everyday challenges.

“I think ZMLA is addressing what I would call the core problem from my perspective, from what we see in health sector, because the challenge really revolves around leadership and management. It comes down to the most efficient ways of using resources and someone has to have the leadership roles clearly outlined. I think it gives you the tools to break down the problem and see what solutions you can come up with at your level. This course boosts confidence to health workers”.

ZMLA Trainee and mentor

### Human resource performance management

ZMLA training under the human resource module covered in detail the use and application of annual performance appraisal system (APAS). Evaluation findings showed that most managers started applying APAS in their workplaces after ZMLA training.

Before ZMLA training, APAS was viewed as a complex undertaking by virtually all Zambian health officials. After ZMLA training most participants were better equipped to complete the APAS than their non-ZMLA counterparts.

“We are now doing APAS, though not all staff are trained. With the few who were trained through ZMLA, we have been helping others to make individual work plans. There were a number of staff who were not confirmed but now some of them have been confirmed after completing their APAS.”

ZMLA trainee Chongwe

Many trainees who came from districts or provinces where the top managers were not ZMLA trained complained of delays in being appraised and wished their managers had undergone ZMLA training.

“What pains us is that, I have gotten this extra skill but nobody here is able to recognize it…I wish our boss had done ZMLA course, he will appreciate my skills much more.”

ZMLA trainee, Mansa

### Change in meeting culture

Qualitative interviews showed improvements in the meeting culture and a greater appreciation for the importance of meetings. Almost all interviewees confirmed that the manner in which they now conducted meetings had improved after the ZMLA training. The workplace climate survey findings showed that the way meetings were conducted in the workplace was among the top three work environment themes that were most improved post-ZMLA training. Meetings were brief and to the point and included action items and responsible individuals.

“…..We have changed how we hold meetings. What we have started doing is that before a management meeting, we ask for departmental inputs …then set up an agenda with time allocation for each agenda item. If you want something discussed, it has to be sent early so that it is included in the agenda. We also circulate the agenda before each meeting. At the end of the meeting there are action points with responsible people clearly shown… this was not the case before ZMLA.”

ZMLA trainee, Chongwe

### Shared vision, teamwork and coordination at workplaces

Interviewees reported that a shared vision was more emphasized following ZMLA training. Trainees had a greater understanding of their role and how it supported the shared vision. Improvements in management accountability and ability to delegate were also noted.

“…because most of the people am working with right now have also done the training, so when I begin to say ‘ok gentlemen this is what I think we need to do here’ they have an understanding of what exactly I am talking about so it’s easy for everyone to be on board. So because most of us have been trained at the district health office, it’s been smooth sailing so far.”

ZMLA trainee, Nyimba

“One of the things that has changed is [that there is] at least more coordination between the members at the DHO here and more importantly, being able to document whatever we discuss in management and having action points, and following those up. We have reached a point now where almost everyone knows about management and you can easily delegate.”

ZMLA trainee, Nchelenge

Improvement in shared vision, teamwork and coordination seemed to have improved more in work places where the overall manager was trained in ZMLA.

“Training of top leadership was extremely key. It was key because it also made sure that the leadership understands what’s happening. I think it puts the whole system of the health sector into perspective and I think it was really strategic to train PMOs and clinical care specialists ……indeed the people that may not have been trained in the concepts may not really appreciate them.”

ZMLA mentor and trainee, Solwezi

### Improved financial management and accountability in workplaces

ZMLA training had improved financial literacy among trainees. Most trainees interviewed appreciated the role of the accountant and the need to adhere to financial regulations much more.

“The ZMLA training drew participants from all departments. The biggest challenge we had was managing the human resource, the people from different units. As accounts unit, I think we are very knowledgeable with financial regulations, but that was not the case with people from other departments. After the ZMLA training, we are seeing great improvement and compliance to financial regulations; there is now strict adherence to budgets. People here were not very comfortable with the bureaucracy we have in accounts, but after the training they appreciate and they understand the importance of observing these controls.”

ZMLA trainee, Kasama

## Discussion

The study found that the ZMLA course improved individual knowledge and skills in leadership and management as demonstrated through individual score change before and after the course in various domains. This has implications for health system performance in the short and long term.

Overall, the assumptions of the ZMLA theoretical framework were supported by the results. The model suggested that through exposure to programme activities (workshops, mentoring, case studies), programme participants should demonstrate increased knowledge, skills, and confidence related to management and leadership functions, and experience improved job motivation, which should in turn affect their behaviour and health outcomes. There was evidence to suggest that exposure to the programme resulted in increased individual knowledge, skills and motivation, with most participants showing confidence in leadership and management practices. There was also a positive impact at workplaces with ZMLA trainees. There was some indication that service delivery also showed improvement, though this was not fully evaluated in this study. This shows that the ZMLA conceptual framework could be a useful model for analysing implementation processes and outcomes for courses aimed at improving leadership and management practices for health workers in low-income settings. This is an extremely important finding and provide opportunities for incremental knowledge gain over time especially in the context of health system strengthening where assessing of leadership and management remain poor despite the urgent need to improve how health systems are governed and managed to improve health outcomes.

Four characteristics distinguished the ZMLA course. These included the short course duration, team based training, mentorship and being a practical/hands on course. The ZMLA course was said to be different from other management courses in that it was done over a short period of time with less disruption to health services. The course was run for 6–12 months from the beginning to the end. Participants were away from their workplaces for a very short time, thus ensuring continuity of services. This was in contrast to the other courses which take up to 18 months with health workers being required to take full study leave, thus negatively affecting service delivery in districts. This course is therefore more acceptable and feasible in low-income settings were human resource challenges exist.

One key success factor to the ZMLA approach was team-focused training, where team members from the same district or health facility were invited for training at the same time and encouraged to work on problems related to their workplace in applying some of the principles learnt during the course. Literature has reported the benefit of team-based leadership training. Such an approach has been shown to leverage different strengths of team members and allow for team building, which is critical in organisation performance[14]. This was evident with trainees who participated in the ZMLA course, where respondents found team training beneficial in helping them to bond and discuss openly the challenges faced at their own institutions and coming up with interventions, which were linked to group case studies and was a requirement to complete the course. Strongly related to team training was the importance of having a team leader at the targeted institution taking part in the training. This was reported to be a key enabler in adopting and applying principles learned through the course. Places where the team leaders were left out of training struggled to implement what was learnt in leadership training due to resistance from top management who did not appreciate the new principles and approaches taught in ZMLA. Other studies have supported the need to include top leadership in such trainings [15].

It was obvious that the ZMLA course had made a difference for individual participants and in some places there was evidence to suggest that the work environment had changed. Some of the changes that were evident in the workplaces included the culture of holding meetings, application of Annual Perfomance Apprasail System (APAS) in the workplace, delegation of responsibility and more constructive feedback/better communication. Almost all respondents confirmed that the meetings were conducted meetings had improved after the ZMLA training. The meeting culture had changed with many more appreciating the importance of meetings. Meetings were now regularly scheduled and agenda items and minutes were sent in advance. One item that was dropped from almost all agendas as a result of exposure to ZMLA was “any other business”. This was found to be one of the reasons why many meetings were unpredictable and lasting for several hours. In terms of actual conduct of meetings, it was reported that now meetings were brief and to the point with clear action points and responsibilities assigned. This saved a lot of time, which in the past would be spent attending endless meetings. Similar findings have been reported in Gambia where both individual management skills and organisation performance improved following implementation of a similar course[16]. In health systems where health workers are expected to perform management duties and attend to patients, a course like ZMLA could be very important in ensuring better time management with better outcomes for the health system and patients.

The Ministry of Health recently introduced annual performance appraisal system (APAS) and many provinces and districts were oriented on the use of APAS before undergoing ZMLA training. Nonetheless, the application of APAS in the workplaces remained very low. Interestingly, most participants admitted that ZMLA had simplified the understanding of APAS, which many were struggling to master. Most participants who had done ZMLA were able to make individual work plans for appraisal and were also appraising those working under them. Application of APAS in the workplace was less evident if the top manager or team leader had not attended ZMLA training. Many participants who came from districts or provinces where the top managers were not ZMLA trained complained of delays in being appraised and wished their managers were also ZMLA trained.

Mentorship was a critical component of ZMLA training with all trainees being allocated mentors who would follow them up throughout the duration of the course, helping to think through challenges of applying lessons learnt and conducting case studies. Despite some few challenges, participants appreciated this approach. This is in line with what has recently been reported in literature that while didactic educational processes can be useful for providing ideas, material and motivation, it is not sufficient [1]. The extent to which leadership and management get integrated into the personal identity depends on the extent to which the skills and knowledge are integrated into daily practice. This should be reinforced through coaching and mentoring [1]. Mentorship formed a basis for trust and on-going support beyond the duration of the ZMLA course.

According to the ZMLA framework, the learning and application of ZMLA principles should translate into not only improved individual knowledge and work environment, but also lead to improved service delivery in terms of quality and coverage. We used selected case studies to evaluate the impact of ZMLA training on some services (Data not shown). Though there was some suggestion that some services improved following implementation of ZMLA case studies in some places, attribution of such effect to ZMLA remained a challenge. Firstly, the implementation of ZMLA case studies and principles was not done in isolation. There were many other activities which were going on in many sites and there were other partners who were targeting similar services. Other limitations in attribution were that the HMIS data was very poor in many sites. In addition, the time to observe changes in service delivery was too short. The challenge of linking leadership and management training to health outcomes has been reported in literature. Rigorous study designs that allow sufficient time to establish linkages between leadership training and health outcomes are being advocated [4, 8].

We explored the reasons why ZMLA was so popular and special among participants. One reason given across all cadre categories was that ZMLA was filling an important gap in leadership and management knowledge and skills among health workers. The course was said to increase self-confidence in leaders and provided them with practical tools to address everyday challenges. The course was specially tailored to the health sector and timed to minimise disruption to health services. The fact that the course was officially certified by the National Institute for Public Administration (NIPA), made it even more valuable for individual participants. They admitted that they could use not only the knowledge gained, but also the diploma to apply for promotion or other jobs. The popularity of the course among other health workers who had not undergone ZMLA training increased when they saw colleagues getting diplomas. Even those who had dropped out of the course in phase I came back to complete the course when they realised that they would get a diploma at the end of the ZMLA course. This finding emphasised the need for accreditation of such courses especially if they are targeted at busy health workers who may not be willing to attend non-recognised courses.

While the ZMLA course had many positive attributes, it also had challenges. Some of these were improved upon in the second and third phases while other challenges still remained. Generally, participants agreed that the timing for the ZMLA course was too short. Many participants struggled to work through group and individual case studies, while others felt some components of the theory were rushed and there was little time for group work and practical sessions. One component of the course that was poorly organised was mentorship after completing the course work.

Interestingly, the graduation of participants was only based on course attendance and writing a case study proposal, but not implementation of the case study. This contributed to failure to implement case studies by many ZMLA trainees. Nonetheless, it was felt by many respondents that implementation of case studies was crucial in ensuring trainees actually translated the theory into everyday practice.

This study looked at ways in which the ZMLA course could be made sustainable. It was clear from the responses that most participants wanted ZMLA to continue in its current form and even extended to other cadres at health facility and community levels. Unfortunately, the course was marketed as a free donor-supported programme. In addition, most health workers expected to be paid allowances for attending the course. Interestingly, most respondents admitted that the way the course was run was expensive and was not sustainable. It was suggested that if the course were institutionalised by the University of Zambia or similar institution, this would make it cheaper and more sustainable.

The study had several limitations: The study used purposeful sampling to identify respondents and qualitative methods were used to collect information from key informants. This limits the generalizability of our findings as the sample might not be representative. We triangulated data sources to improve validity of the results. The self-reporting responses which were used in the pre-test and post-tests quizzes, workplace climate and leadership and management tools might be affected by a tendency to report desired results and selection of higher scores. This was probable as noted in the scores, which tended to be generally high pointing to the ceiling effect in the instrument we used. However, it was explained to the respondents that they were to be honest with answers given. The study design did not include a comparison group and there was no counterfactual to compare the effect of ZMLA. It was therefore not possible to attribute the effect on performance, such as change in service delivery to ZMLA as there were other confounders that could not be controlled for.

## Conclusion

The study showed that the ZMLA course was well appreciated by individual trainees and stakeholders. The course was seen by many as a motivator, a source of skills and knowledge, and a tool that empowered health system managers to understand their roles and challenges. Evidently, the course had improved leadership and management knowledge and skills for targeted public healthcare workers. There was evidence suggesting that workplace climate had improved in sites with ZMLA trainees.
